# Helmet continuous positive airway pressure versus high-flow nasal cannula in COVID-19: a pragmatic randomised clinical trial (COVID HELMET)

**DOI:** 10.1186/s13063-020-04863-5

**Published:** 2020-12-03

**Authors:** Jonas Tverring, Anna Åkesson, Niklas Nielsen

**Affiliations:** Lunds universitet Medicinska fakulteten, Lund, Sweden

**Keywords:** COVID-19, Randomised controlled trial protocol, Helmet CPAP, HFNC, Ventilator-free days

## Abstract

**Background:**

Patients with COVID-19 and hypoxaemia despite conventional low-flow oxygen therapy are often treated with high-flow nasal cannula (HFNC) in line with international guidelines. Oxygen delivery by helmet continuous positive airway pressure (CPAP) is a feasible option that enables a higher positive end-expiratory pressure (PEEP) and may theoretically reduce the need for intubation compared to HFNC but direct comparative evidence is lacking.

**Methods:**

We plan to perform an investigator-initiated, pragmatic, randomised trial at an intermediate-level COVID-19 cohort ward in Helsingborg Hospital, southern Sweden. We have estimated a required sample size of 120 patients randomised 1:1 to HFNC or Helmet CPAP to achieve 90% power to detect superiority at a 0.05 significance level regarding the primary outcome of ventilator free days (VFD) within 28 days using a Mann-Whitney *U* test. Patient recruitment is planned to being June 2020 and be completed in the first half of 2021.

**Discussion:**

We hypothesise that the use of Helmet CPAP will reduce the need for invasive mechanical ventilation compared to the use of HFNC without having a negative effect on survival. This could have important implications during the current COVID-19 epidemic.

**Trial registration:**

ClinicalTrials.gov NCT04395807. Registered on 20 May 2020

## Administrative information

**Table:** 

Title {1}	Helmet Continuous Positive Airway Pressure versus High-Flow Nasal Cannula in COVID-19: A pragmatic randomised clinical trial (COVID HELMET)
Trial registration {2a and 2b}.	Clinicaltrials.gov, NCT04395807
Protocol version {3}	Version 1.0, 18 June 2020
Funding {4}	This trial is investigator-initiated and no specific funding was received. The sponsor purchased the medical devices used in this trial in the context of state-funded healthcare and not for the main purpose of use in a trial. The sponsor also paid for the application for ethical approval. The principal investigator (JT) have previously received funding from Swedish Government Research Grant (ALF) and the senior investigator (NN) have previously received funding from The Swedish Research Council and the Swedish Heart and Lung Foundation. The study statistician (AÅ) is funded by the sponsor.
Author details {5a}	Jonas Tverring^1,2^ 1. Department of Infectious diseases, Helsingborg Hospital, Region Skåne, Sweden 2. Division of Infection Medicine, Department of Clinical Sciences, Faculty of Medicine, Lund University, Sweden Niklas Nielsen^3,4^ 3. Department of Anaesthesia and Intensive Care, Helsingborg Hospital, Region Skåne, Sweden 4. Division of Anaesthesiology and Intensive Care, Department of Clinical Sciences, Faculty of Medicine, Lund University, Sweden Anna Åkesson^5^ 5. Clinical Studies Sweden – Forum South, Skåne University Hospital, Lund, Sweden
Name and contact information for the trial sponsor {5b}	Region Skåne, Skånes sjukhus Nordväst, Helsingborg Hospital Södra Vallgatan 5, 251 87 Helsingborg, Sweden +46 42 406 10 00 Academic sponsor: Lund University, Faculty of Medicine, Sweden
Role of sponsor {5c}	The sponsor had no participation or authority in the study design or writing of the protocol or the decision to submit the report for publication. The study is performed entirely independently from manufacturers of the HFNC and Helmet CPAP devices.

## Introduction

### Background and rationale {6a}

Patients requiring hospitalisation for COVID-19 predominantly present with acute hypoxaemic respiratory failure (AHRF) [1]. Conventional low-flow oxygen therapy will suffice for patients with mild to moderate disease but patients with severe or critical illness will require more advanced support [2]. Endotracheal intubation and mechanical ventilation constitute the highest level of care for patients with AHRF but beds and ventilators in the intensive care unit (ICU) are limited. High-flow oxygen therapy through a nasal cannula (HFNC) is a mainstay of treatment in patients with COVID-19 failing on conventional oxygen therapy in our institution and has recently been endorsed in the surviving sepsis campaign (SSC) guidelines on COVID-19 [3]. HFNC have been shown to improve comfort, oxygenation and 90-day survival, but not intubation rates, compared to standard low-flow oxygen therapy in patients with AHRF [4]. Non-invasive ventilation (NIV) using a continuous positive airway pressure (CPAP) should theoretically improve lung aeration and gas exchange in AHRF compared to HFNC because it enables a higher positive end expiratory pressure (PEEP), similar to that of mechanical ventilation [5]. Yet, NIV is receiving mixed support in relation to COVID-19 [3]. This originates from some observational studies that have reported high failure rates and associated high hospital mortality when using conventional CPAP face masks where tolerance is a well-known issue [6, 7]. In comparison to masks, Helmet CPAP show superior tolerance, significantly reduced intubation rates and improved survival in AHRF and acute respiratory distress syndrome (ARDS) respectively [8, 9]. A systematic review from 2017 found a significant reduction in hospital mortality from using Helmet CPAP in AHRF but stated that there was insufficient evidence to make clear recommendations [10]. The SSC guidelines on COVID-19 make a similar conclusion, positioning the Helmet CPAP as a feasible option for patients with COVID-19 failing on conventional oxygen therapy, but authors were unable to make specific recommendations due to a lack of direct evidence [3]. Helmet CPAP have been used extensively to treat COVID-19 in northern Italy with anecdotally good results [11]. It is our hypothesis that the use of Helmet CPAP will reduce the need for intubation in patients with AHRF from COVID-19 compared to the use of HFNC, without having a negative effect on survival.

## Objectives {7}

### Primary objective

To compare the performance of Helmet CPAP versus HFNC regarding the number of ventilator free days (VFD) within 28 days in patients with COVID-19 and AHRF.

Secondary objectives:

- To evaluate oxygen delivery efficacy after 1 h on the Helmet CPAP versus HFNC
- To compare the level of patient-reported comfort after using the Helmet CPAP versus HFNC at 24 h from randomisation
- To investigate the relative incidence of carbon dioxide rebreathing in patients using Helmet CPAP versus HFNC
- To compare the short-term (28 days) and longer-term (180 days) all-cause mortality in patients randomised to Helmet CPAP versus HFNC
- To compare the frequency of intubation in patients randomised to Helmet CPAP versus HFNC

## Trial design {8}

Investigator-initiated, randomised, controlled, investigator-blinded, pragmatic superiority trial with a parallel group design, 1:1 concealed allocation ratio and a 28-day main follow-up period.

## Methods: participants, interventions and outcomes

### Study setting {9}

A medical emergency care ward functioning as an intermediate-level COVID-19-cohort in Helsingborg hospital, Region Skåne, Sweden. The hospital is a teaching emergency hospital that serves a population of approximately 250,000. Helsingborg Hospital is a suitable institution for this trial because the hospital has both the Helmet CPAP and HFNC in regular use and southern Sweden is still expecting its COVID-19 epidemic peak.

### Eligibility criteria {10}

Patients will be screened for eligibility by the attending ward physician (specialist in internal medicine) using the following criteria:

Inclusion criteria (all of the following):

- Age ≥ 18 years
- Sars-Cov-2 found in the respiratory tract by PCR during the current disease episode
- Peripheral oxygen saturation (SpO2) < 92% *despite* conventional low-flow oxygen therapy of at least 6 L/min for at least 15 min
- A decision to initiate HFNC or Helmet CPAP by the attending ward physician
- The patient has given written informed consent to participate

Exclusion criteria (any of the following):

- Need for direct admission to the intensive care unit for mechanical ventilation
- Unconsciousness or drowsiness
- Pneumothorax
- Carbon dioxide pressure (pCO2) > 6 kPa in venous blood gas (VBG)
- Underlying chronic obstructive pulmonary disease stage III-IV
- A decision not to participate
- Inability to comprehend the study content and give informed consent

### Who will take informed consent? {26a}

The attending ward physician (a specialist in internal medicine) will be responsible for obtaining informed consent from trial participants prior to randomisation. The physician will provide oral and written information about the study, any risks or benefits involved with participation and inform that termination from the study can be done at any time without explanation in accordance with the ethical approval. Patient information will be provided as early as possible when patients are admitted to the intermediate-level COVID-19 cohort ward and generally when they are still on low-flow oxygen therapy to allow adequate time to make an informed decision.

### Additional consent provisions for collection and use of participant data and biological specimens {26b}

The data collected for this study may be used in ancillary studies, but we have no plans to collect additional participant data or biological specimens outside of what is mentioned in this protocol.

## Interventions

### Explanation for the choice of comparators {6b}

HFNC is endorsed as the standard treatment for patients with COVID-19 failing on conventional low-flow oxygen therapy (i.e. the target study population) in both local treatment guidelines and current international guidelines [3].

### Intervention description {11a}

#### Intervention

Helmet CPAP (CaStar hood for CPAP therapy by Starmed/Intersurgical) driven by high-flow blender (Bio-Med Devices).

#### Control

HFNC (Optiflow™ nasal high-flow interface) driven by AIRVO 2 humidification system (Fisher and Paykel).

Interventions will be initiated at the time of randomisation and both the Helmet CPAP and HFNC will be applied and titrated according to local best standard of care (SOC). Hereby, HFNC will be started at a flow of 30 L/min with a possibility to increase flow to maximum 60 L/min at the choice of the attending physician (corresponding PEEP ~ 3–5 cmH_2_O [12]). Helmet CPAP will be started at a flow of 40 L/min and a PEEP of 5 cmH_2_O which can be increased to a maximum of 20 cmH_2_O at the attending physician’s discretion. We suspect that the PEEP will normally be used at around 8–10 (− 12) cmH_2_O. The study provides no guidelines on fraction of inspired oxygen (FiO2%) or litres of oxygen per minute. Oxygen settings will be titrated according to the attending physicians’ choice. Local SOC guidelines recommend a target SpO2 of 92%. When patients allocated to the intervention needs to take off the helmet for short periods of time (e.g. for meals), they will generally be put on HFNC according to physician’s choice. Included patients who require transportation from the medical COVID-cohort to the ICU for mechanical ventilation will be put on a portable HFNC during transportation between wards and prior to intubation according to the wishes of our local intensivists. Extubated patients will also be put on HFNC regardless of treatment allocation according to the wishes of our local intensivists. We believe that these circumstances will only have a moderate effect on the primary outcome because we expect the time from transportation to intubation to be short and we expect the number re-intubations within 28 days to be few.

### Criteria for discontinuing or modifying allocated interventions {11b}

There are no mandated criteria for discontinuing or modifying allocated interventions, but the patient and the treating physician may choose to do so at any time, upon which they will be asked to report timing and reason for non-adherence. Patients moving to the ICU due to deterioration or to a regular ward due to clinical improvement will be able to adhere to the standard protocol follow-up. In the (improbable) event of a patient being moved while having an unchanged need for respiratory support to another hospital or ward that does not have the treatment device available allocated to the patient (i.e. Helmet CPAP), we will regard this as an allocation violation, but we should still be able to assess the primary outcome on an “as randomised”-basis. Patients on the Helmet CPAP will necessarily have to take off the Helmet for shorter periods of time during meals. We will actively register the number of hours spent in each device each day on a case report form (CRF) as a continuous evaluation of allocation adherence. More than 6 h per 24 h off the allocated device will be registered as an allocation deviation.

### Strategies to improve adherence to interventions {11c}

The Helmet CPAP can be associated with discomfort from bad fit and airflow noise. Patients will be provided with earplugs and we will carefully select strap system and helmet size (XS, S, M, L or XL) to ensure optimal fit for each patient. High airflow levels in HFNC can be a discomfort which is why we recommend a starting flow of 30 L/ min and allow titrations at the choice of the treating physician in consultation with the patient.

### Relevant concomitant care permitted or prohibited during the trial {11d}

We make no restrictions on concomitant care or interventions during the trial.

### Provisions for post-trial care {30}

We have no plan for ancillary or post-trial care or compensation for the participants. This is a pragmatic trial comparing two current standards of care.

### Outcomes {12}

#### Primary outcome

Ventilator-free days (VFD) within 28 days after randomisation. Patients who die within 28 days will be counted as 0 VFD. Time in ventilator will be counted in hours and rounded to whole days (continuous data).

Secondary outcomes:

- SpO2/FiO2 ratio assessed 1 h after randomisation
- Patient comfort 1–10 (visual analogue scale) assessed 24 h after randomisation
- Frequency of endotracheal intubation within 28 days from randomisation
- Frequency of carbon dioxide rebreathing (pCO2 > 6 kPa in a VBG) within 28 days from randomisation
- Days alive within 28 days from randomisation
- Days alive within 180 days after randomisation (not in primary publication)

#### Outcomes rational

Based on the best current evidence [4, 9], we expect any difference in efficacy between intervention and control to be due to a difference in intubation rate and/or possibly time in respirator rather than survival. Respirator use is a particularly clinically relevant outcome during the current COVID-19 pandemic when ICU beds are scarce. The benefit of choosing VFD as a primary outcome rather than intubation frequency is that VFD strongly penalises death. Hereby, VFD effectively assesses our primary concern for harm related to the intervention, namely, an adverse delay to intubation leading to reduced survival. The drawback of VFD is a potential dilution of harm (death) due to high efficacy (intubation rate) which is why we are also including days alive within 28 and 180 days as secondary outcomes. The 180 days mortality outcome will not be reported in the primary manuscript because we do not wish to delay dissemination of findings, but we consider it necessary to evaluate since many patients with COVID-19 have prolonged hospital stay. SpO2/FiO2 ratio is included as a sort of proof of concept outcome with the purpose of confirming or rejecting our underlying assumption that the higher PEEP enabled by the Helmet CPAP will improve lung aeration. We also have a patient-centred outcome regarding self-reported comfort. Both devices are reported to have superior tolerance compared to low-flow oxygen therapy and CPAP masks but they have never been tested head-to-head in adults. Device comfort may be particularly important if the trial does not show superiority or if the effect size is small. Lastly, we have included a secondary outcome on carbon dioxide rebreathing which, according to our judgement, is the main harm apart from death and comfort/tolerance issues that can be considered more likely for the intervention group than the control group. We are taking measure to avoid this from happening (i.e. minimum Helmet CPAP air flow 40 L/min).

### Participant timeline {13}

**Fig. 1:**
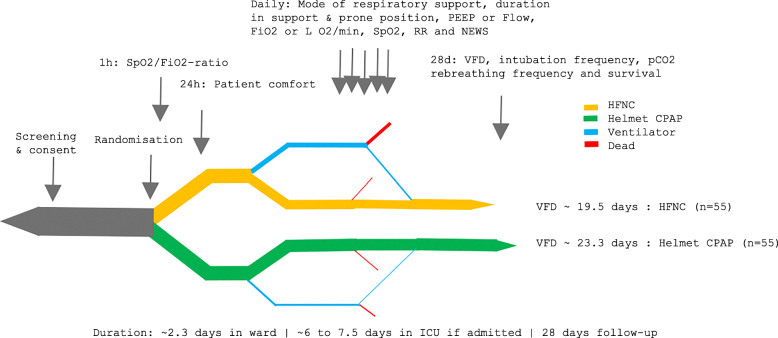
Participant timeline. Patients in the medical intermediate COVID-19 cohort ward will be screened for eligibility according to the inclusion and exclusion criteria, given written and oral information and then asked for written informed consent. Secondary endpoints will be collected at 1 h, 24 h and 28 days from randomisation, when the primary endpoint of ventilator-free days will also be assessed. We plan to include 60 patients in each group and expect the HFNC group to have ~ 19.5 VFD and the Helmet CPAP group to have ~ 23.3 VFD at 28 days. The thickness of the lines corresponds to the expected patient density in intervention versus control group and treatment in ward versus intensive care and in patients who survive or die within 28 days from randomisation. Abbreviations: SpO2, peripheral oxygen saturation; FiO2 ratio, fractional inspired oxygen concentration; RR, respiratory rate; PEEP, positive expiratory end-pressure; NEWS, national early warning score; AE, adverse events; VFD, ventilator-free days (within 28 days); ICU, intensive care unit; CPAP, continuous positive airway pressure

Please see Fig. 1 for the participant timeline.

### Sample size {14}

We have estimated a required sample size of 120 patients (60 per group) to achieve 90% power to detect superiority at a 0.05 significance level. This estimate is based on the following clinical assumptions and statistical calculations:

1. A median of 1 out of 3 patients started on HFNC or Helmet CPAP will be started on invasive mechanical ventilation [2].
2. Patients put on the ventilator will be treated in the ward for median 2.3 days before intubation, and ventilated patients will have 67.4% ICU mortality, and those who survive will stay on the ventilator for a median of 6 days [13].
3. Patients not put on the ventilator will have 8.1% 28-day mortality and survive for a median of 7.5 days [2].
4. The Helmet CPAP will avoid 3 out of 5 intubations compared to HFNC, which is an estimate based on (A) Frat et al. [4] who reported 40/106 (38%) intubation frequency in HFNC treated patients with AHRF versus 55/110 (50%) in patients with CPAP masks (*p* = 0.18 for comparison) and (B) Patel et al. [9] who reported 24/39 (61.5%) intubation frequency in patients with ARDS treated with CPAP mask versus 8/44 (18.2%) treated with Helmet CPAP (*p* < 0.001 for comparison). Hence, we expect treatment with Helmet CPAP to require (18.2/61.5)/(38/50) ≈ 2 out of 5 intubations compared to HFNC.

This leads to an estimated VFD of 19.545 for patients on HFNC (calculation: ((2/3*0.919*28) + (1/3*0.326*22))) and 23.257 for patients on Helmet CPAP (calculation: ((13/15*0.919*28) + (2/15*0.326*22))). We assume the standard deviation (SD) to be 6 in both groups based on a mean estimate of SDs reported from five trials with VFD as the primary endpoint in the paper by Yehya et al. [14]. Because we expect a reduction of intubation frequency and days in ventilator to be the driving factor behind a reduction in VFD rather than mortality, we will use Mann-Whitney *U* test as the primary statistical method, also based on argumentation by Yehya et al. [14]. In order to estimate the sample size, data was simulated with mean 23.257 for the intervention group and mean 19.545 for the control group, the standard deviation was set to 6 for both groups, and then the sample size was varied by simulation until the proportion of significant (0.05) two-sided Mann-Whitney *U* test was equal to the desired power (0.90) using R statistical software (See Additional file 1 for R code). This resulted in a required sample size of 60 patients per group.

### Recruitment {15}

Clinical investigator Leif Angelison MD PhD student, Helsingborg Hospital, Sweden, will be principally responsible for overseeing the study implementation on the floor. Information on the study eligibility criteria have been orally presented to physicians working in the medical department at a morning meeting. Information to study nurses has been disseminated both in written form by email and presented at three times to cover all staff working in the ward. Written and oral information have also been presented to ICU physicians at one meeting. Written and oral information about the study and eligibility criteria will be repeatedly presented to involved health-care workers to ensure a high percentage of enrolment. We will keep a dedicated screening log of all patients treated in the ward, patients who received the patient information, patients who accepted participation and patients who reached the inclusion criteria during their hospital stay, whether they were included in the study or not. We consider recruitment of 120 patients in our hospital to be feasible based on the COVID-19 epidemiological estimates for the Skåne Region from the Public Health Agency of Sweden [15].

## Assignment of interventions: allocation

### Sequence generation {16a}

We have generated a blocked random number sequence without stratification using StataMP 16.1 and the Stata module Ralloc [16]. Stata code for the randomisation sequence can be provided on request.

### Concealment mechanism {16b}

The allocation sequence is implemented in REDCap’s Internet-based randomisation application [17] and concealed within REDCap until interventions are assigned.

### Implementation {16c}

The trial investigator will generate the allocation sequence and lock the sequence within REDCap. The attending ward physician will screen patients and ask for informed consent. A study nurse will assign participants to interventions. REDCap uses a two-step verification for log-in and only study nurses who are eligible for including patients will have access to username and password. They will not have access to the allocation sequence or block size.

## Assignment of interventions: blinding

### Who will be blinded {17a}

The patients and physicians, nurses and other healthcare workers involved with direct patient care will not be blinded to allocation group due to the inherent difficulty in blinding the intervention. The author group (JT and NN) and statistician (AÅ) will be blinded to group allocation. When data is extracted from the REDCap database, the allocation will be coded as “0” and “1” and the Code Key will only be revealed after statistical evaluation of the primary and secondary endpoints.

### Procedure for unblinding if needed {17b}

Not applicable since patients and healthcare workers are not blinded to the allocation.

## Data collection and management

### Plans for assessment and collection of outcomes {18a}

In order to keep the trial pragmatic during the ongoing epidemic, we have chosen to keep prospectively collected data to a minimum, while still ensuring validity in the primary and secondary outcomes. We have chosen to use an electronic case report form (eCRF) at screening and randomisation to allow secure concealment, allocation and data registration with data range checks. But in order to be consistent with the strict hygienic rules associated with the care of COVID-19 patients, we will use a paper-based CRF for prospective daily data collection. CRFs can be sent on request. We plan to collect National early warning score (NEWS) and Clinical frailty index (CFS) as baseline predictors of disease severity. NEWS2 **≥** 5 had 87% sensitivity (95% CI 60 to 98%) and 71% specificity (95% CI 56 to 83%) to predict severe disease from COVID-19 in a recent study (*n* = 66) [18]. CFS > 5 was recently shown to double the risk for in-hospital mortality (HR 1.93, 95% CI 1.02 to 3.65) in 250 patients with COVID-19 [19]. We will use a visual analogue scale (VAS) to measure patient comfort. VAS has been validated previously concerning comfort perception of footwear [20]. See Table 1 data collection flow chart below for details on timing, method and specification of data collected.

### Plans to promote participant retention and complete follow-up {18b}

We consider loss-to-follow-up unlikely for this trial. Most outcomes include events that are compulsorily registered in electronic health records and can be easily accessed retrospectively. The exception is the secondary outcomes of SpO2/FiO2-ratio and patient comfort which requires active patient participation, but these outcomes are assessed early in the study (at 1 h and 24 h from randomisation respectively). Participants who deviate from the intervention protocol will still be evaluable “as-randomised” for the primary outcome as well as for the secondary outcomes of intubation frequency and 28-day and 180-day survival. However, if patients chose to discontinue the trial and do not wish to participate in such assessment, any collected data will be discarded, and the participant will only appear in the study flow chart. Patients who are included in the trial but dismissed from hospital before the main follow-up period of 28 days have passed will be contacted by phone by a trial investigator to ensure that they have indeed been alive and out of respirator during the remaining days up until 28 days after randomisation.

### Data management {19}

Screening and baseline data will be entered live using an eCRF at the time of randomisation. Further data on primary and secondary outcomes will be entered into the REDCap database collectively after all patient recruitment has finished, using filled-out paper CRFs and electronic health records. Individual patient data will be handled as ordinary chart records and will be kept according to the European General Data Protection Regulation (GDPR). All original records (e.g. consent forms and CRFs) will be retained at the Department of Infectious Disease in Helsingborg Hospital for 15 years to allow inspection by relevant authorities. The coded trial database will be maintained for 15 years if requested for revision.

**Table 1:** Data collection flow chart

Timing	Method	Data type and specification
At screening	eCRF (RedCap)	Inclusion and exclusion criteria and informed consent
At randomisation	eCRF (RedCap)	Allocation, treatment limitations (i.e. ICU admission), weight, height, place of birth, smoking, date of symptom start, gender, clinical frailty scale, mode of respiratory support, SpO2%, litres O2/min, RR/min and NEWS
One hour post randomisation	CRF paper	SpO2/FiO2-ratio
24 h post randomisation	CRF paper	Patient comfort (visual analogue scale (VAS) 1–10)
Daily for 28 days	CRF paper	Mode of respiratory support, number of hours per 24 h in allocated support, nr of hours in prone position per 24 h, NEWS, PEEP or Flow, SpO2%, RR/min and events with commentary (e.g. allocation violation, death, exit from study, ICU admission or hospital discharge)
Retrospectively from electronic medical charts	eCRF (RedCap)	Comorbidities, vitals, medications, radiology, microbiology and laboratory parameters at triage, randomisation and during hospital stay, and, duration of hospital stay, duration of ICU stay, duration of mechanical ventilation and days alive within 28 and 180 days, respectively.

*Abbreviations*: *eCRF* electronic case report form, *ICU* intensive care unit, *SpO2%* peripheral oxygen saturation, *L O2/min* litres of oxygen per minute, *RR* respiratory rate, *NEWS* national early warning score, *FiO2* fraction of inspired oxygen, *PEEP* positive end-expiratory pressure

### Confidentiality {27}

At the time of randomisation, participants will be assigned a trial ID number which will be used on all the personal information containing documents (e.g. paper CRF and eCRF). The Code Key will be kept in a safe where only the trial investigators have access. The coded information will be kept in an electronic database or in a folder in a locked document cabinet. When data from the study is published, personal data will not be identifiable.

### Plans for collection, laboratory evaluation and storage of biological specimens for genetic or molecular analysis in this trial/future use {33}

We have no plans to collect or store biological specimens in the current or future ancillary studies.

## Statistical methods

### Statistical methods for primary and secondary outcomes {20a}

#### Primary outcome

We will analyse VFD as a continuous outcome between two groups with a non-normal distribution using the Mann Whitney *U* test. We will provide the absolute median difference in VFD between groups with 95% confidence intervals (CI). We will also calculate and present the Mann-Whitney (ϕ) parameter with 95% CI using the R function wmwTest in the asht R package as suggested by Fay et al. [21]. We will also perform a sensitivity analysis of the primary outcome using unadjusted competing risks regression [14]. We hypothesise that the intervention group (Helmet CPAP) will have a significantly (*p* < 0.05) higher VFD than the control group (HFNC).

#### Secondary outcomes

SpO2/FiO2 ratio (a) and patient comfort (b) will also be analysed as continuous outcomes between two groups with the Mann Whitney *U* test. Frequency of endotracheal intubation (c) and carbon dioxide rebreathing (d) are dichotomized outcomes between two groups and will be analysed using a chi-squared test (unless groups are less than 5, then we will use Fisher’s exact test). Days alive within 28 (e) and 180 days (f) from randomisation will be visualised using a Kaplan-Meier plot and analysed using unadjusted Cox regression. We will test proportional hazards assumption using a global test, Schoenfeld residuals, and a log-log plot of survival. We will use Stata MP 16.1 statistical software.

### Interim analyses {21b}

Due to the moderate size of the trial and because we are pragmatically evaluating conventional therapies in current use in our healthcare system, no interim analysis is planned.

### Methods for additional analyses (e.g. subgroup analyses) {20b}

Because baseline characteristics may be skewed between the two groups despite randomisation, we will also perform adjusted analyses for (a) the primary outcome of VFD using competing risks regression and (b) for the secondary outcome of 28- or 180-day survival using Cox regression. Since we are expecting only ~ 26 events (calculation: 60*(1/3*0.674 + 2/3*0.081) + 60*(2/15*0.674 + 13/15*0.081)), we are limited to few co-variates to avoid overfitting. We plan to adjust for age (cont.), Clinical Frailty Index (cont.) and NEWS score at randomisation (cont.). If other baseline characteristics appear severely skewed instead, we may consider adjusting for other co-variates, such as gender, comorbidities (Charlson comorbidity index), BMI, smoking, Nordic origin of birth or serum biomarkers (e.g. lactate, serum D-dimer, lymphocyte count).

### Methods in analysis to handle protocol non-adherence and any statistical methods to handle missing data {20c}

The analyses of the primary and secondary outcomes will be analysed as randomised, regardless of protocol adherence. Analyses according to adherence will be considered if important deviations from the protocol compromise the validity of the “as randomised” analysis. All variables will be screened for frequency and type of missingness (i.e. missing at random or not). Multiple imputation will be used if missingness is above 5% in any variable. In the case of missing data and imputation, complete case analysis will be performed as a sensitivity analysis.

### Plans to give access to the full protocol, participant-level data and statistical code {31c}

We plan to publish the statistical code as a supplement to the main manuscript publication. Participant-level data can be accessed from the principal trial investigator on reasonable request.

## Oversight and monitoring

### Composition of the coordinating centre and trial steering committee {5d}

Jonas Tverring (JT) as the principal investigator is responsible for preparing and revising the protocol and disseminating any changes. JT is also responsible for coordinating data collection and analyses and writing of the scientific manuscript. Niklas Nielsen (NN) as a senior investigator is responsible for overseeing the study design and protocol and interpretation of the findings. Anna Åkesson (AÅ) as a statistician is responsible for overseeing any statistical analyses. Leif Angelison (LA) as the clinical investigator is responsible for overseeing that the study implementation on the floor follows the protocol.

### Composition of the data monitoring committee, its role and reporting structure {21a}

Because this is a pragmatic trial of moderate duration comparing medical devices in daily use, we have not considered there to be a need for a data monitoring committee.

### Adverse event reporting and harms {22}

The Swedish Medical Products Agency (Läkemedelsverket) has reviewed the study design in May 2020 and concluded that it should not be considered a clinical trial in the traditional sense but rather a pragmatic randomised study between two approved medical devices. Hence, reporting of adverse events will not be as ambitious as a clinical trial testing a novel drug. We will aim to evaluate any harm from the intervention in our primary and secondary endpoints on survival, intubation frequency, carbon dioxide rebreathing and patient-reported comfort. There is also a commentary section in the study-specific CRFs where study nurses can report allocation violations or any unexpected side-effects from allocated intervention.

### Frequency and plans for auditing trial conduct {23}

We have no plans for auditing trial conduct in this investigator-initiated pragmatic trial.

### Plans for communicating important protocol amendments to relevant parties (e.g. trial participants, ethical committees) {25}

The principal investigator is responsible for communicating protocol modifications. This will happen firstly to the clinical investigator on the floor (LA) who, together with his medical colleagues, is responsible for screening patients and asking for informed consent, and secondly, to the study nurses who randomise patients. This information will be provided by phone and a physical meeting between the principal and clinical investigator as well as by e-mail and oral presentation to the study nurses. The senior investigator and study statistician will be informed by phone or email. Relevant information will also be updated in the trial registry.

### Dissemination plans {31a}

We plan to publish the trial results in a peer-reviewed medical journal.

## Discussion

We had three practical concerns during the planning stages of the trial in April and May 2020. First was the adequate supply of Helmet CPAP devices. Our local supplier’s stock was small and there was an export ban from Italian manufacturers during April. The ban was eventually lifted on the 11th of May 2020. Second was the feasibility of prone position in patients on the Helmet CPAP. Awake proning seems to be beneficial in COVID-19 and should optimally be available to both treatment groups [22]. In early May, Italian researchers published data to support the rational and feasibility of combining the Helmet CPAP and prone positioning without reports of harm [23, 24]. Third was the number of COVID-19 patients requiring intermediate-level care in our institution. At the time of writing, local COVID-19 incidence seems to have plateaued at a low to moderate level. This indicates that patient recruitment may take considerably longer than initially predicted from April forecasts [15]. Performing the trial at additional centres would naturally have been of benefit from recruitment and validity aspects. However, no other hospital in our region have prior experience from using the Helmet CPAP and we did not regard international cooperation as feasible, or reasonably quick to arrange, during the ongoing pandemic. The single-centre design and modest sample size is an important limitation and we acknowledge that small but clinically important differences may be missed.

## Trial status

Protocol version 1.0, 18 June 2020

Recruitment began on Wednesday 3 June 2020.

The first patient was recruited on 18 June 2020.

Recruitment is planned to be completed during the first half of 2021.

## Supplementary information

**Additional file 1.**

## Abbreviations

AHRF: Acute hypoxaemic respiratory failure
HFNC: High-flow nasal cannula
NIV: Non-invasive ventilation
PEEP: Positive end expiratory pressure
CPAP: Continuous positive airway pressure
ARDS: Acute respiratory distress syndrome
VBG: Venous blood gas
SpO2: Peripheral oxygen saturation
pCO2: Carbon dioxide partial pressure
VFD: Ventilator-free days
FiO2: Fraction of inspired oxygen
ICU: Intensive care unit
NEWS: National early warning score
eCRF: Electronic case report form
RR: Respiratory rate
