# Supplementary material for: Helmet continuous positive airway pressure versus high-flow nasal cannula in COVID-19: a pragmatic randomised clinical trial (COVID HELMET)
Source: Trials. 2020 Dec 3;21:994. doi: 10.1186/s13063-020-04863-5 (PMC7711053; doi:10.1186/s13063-020-04863-5)
Supplement: Supplementary file 1 — Additional file 1. [file 13063_2020_4863_MOESM1_ESM.pdf]

```
#### COVID HELMET protocol appendix ##

#### Simulate Power for M-H test #####

## Primary outcome: Ventilator-free days (VFD) within 28 days
# RCT with 2 groups. 1:1 allocation ratio
# VFD (cont.) : min 0, max 28.
# Expected VFD: Intervention: 19.545, Control: 23.257.
# Expected SD: 6 for both groups
# Alpha 0.05
# Desired power 0.90

### R code ###
set.seed(20200422)
n=seq(30,70,1)
B=seq(1,10000,1)
temp=matrix(NA, nrow = length(B), ncol = 1)
pow1=data.frame(n=n, power=matrix(NA, nrow = length(n), ncol =
1))
for (i in seq_along(n)) {
  for (j in seq_along(B)) {
    control<-rnorm(n[i], 19.545, 6)
    control[control>28]<-28
    control[control<0]<-0
    intervervention<-rnorm(n[i], 23.257, 6)
    intervervention[intervervention>28]<-28
    intervervention[intervervention<0]<-0
    temp[j]=wilcox.test(control,intervervention)$p.value
  }
  pow1[i,2]=sum(temp<0.05)/length(B)
}
View(pow1)
which(pow1$power>0.9)
pow1[26,1]
### End code ###

# Results: n=60 per group
```
